# Quantitative parameters of enhanced dual-energy computed tomography for differentiating lung cancers from benign lesions in solid pulmonary nodules

**DOI:** 10.3389/fonc.2022.1027985

**Published:** 2022-10-06

**Authors:** Changjiu He, Jieke Liu, Yong Li, Libo Lin, Haomiao Qing, Ling Guo, Shibei Hu, Peng Zhou

**Affiliations:** Department of Radiology, Sichuan Cancer Hospital & Institute, Sichuan Cancer Center, School of Medicine, University of Electronic Science and Technology of China, Chengdu, China

**Keywords:** dual-energy computed tomography, iodine concentration, solid pulmonary nodule, lung cancer, benign lesion

## Abstract

**Objectives:**

This study aimed to investigate the ability of quantitative parameters of dual-energy computed tomography (DECT) and nodule size for differentiation between lung cancers and benign lesions in solid pulmonary nodules.

**Materials and Methods:**

A total of 151 pathologically confirmed solid pulmonary nodules including 78 lung cancers and 73 benign lesions from 147 patients were consecutively and retrospectively enrolled who underwent dual-phase contrast-enhanced DECT. The following features were analyzed: diameter, volume, Lung CT Screening Reporting and Data System (Lung-RADS) categorization, and DECT-derived quantitative parameters including effective atomic number (Zeff), iodine concentration (IC), and normalized iodine concentration (NIC) in arterial and venous phases. Multivariable logistic regression analysis was used to build a combined model. The diagnostic performance was assessed by area under curve (AUC) of receiver operating characteristic curve, sensitivity, and specificity.

**Results:**

The independent factors for differentiating lung cancers from benign solid pulmonary nodules included diameter, Lung-RADS categorization of diameter, volume, Zeff in arterial phase (Zeff_A), IC in arterial phase (IC_A), NIC in arterial phase (NIC_A), Zeff in venous phase (Zeff_V), IC in venous phase (IC_V), and NIC in venous phase (NIC_V) (all *P* < 0.05). The IC_V, NIC_V, and combined model consisting of diameter and NIC_V showed good diagnostic performance with AUCs of 0.891, 0.888, and 0.893, which were superior to the diameter, Lung-RADS categorization of diameter, volume, Zeff_A, and Zeff_V (all *P* < 0.001). The sensitivities of IC_V, NIC_V, and combined model were higher than those of IC_A and NIC_A (all *P* < 0.001). The combined model did not increase the AUCs compared with IC_V (*P* = 0.869) or NIC_V (*P* = 0.633).

**Conclusion:**

The DECT-derived IC_V and NIC_V may be useful in differentiating lung cancers from benign lesions in solid pulmonary nodules.

## Introduction

Lung cancer is the leading cause of cancer-induced death worldwide (1–3). With the popularization of lung cancer screening and computed tomography (CT), the detection rate of pulmonary nodules has been greatly improved (4). Now the Lung CT Screening Reporting and Data System (Lung-RADS) is widely used to assess and manage pulmonary nodules according to the nodule size (5, 6), as the malignancy probability of a given nodule increases with its size (7). However, previous studies demonstrated that the Lung-RADS categorization had insufficient diagnostic accuracy for distinguishing lung cancers from benign lesions appearing as solid pulmonary nodules (8, 9). The relatively low specificity of the Lung-RADS categorization may lead to excessive diagnosis and treatment of benign nodules (9, 10). Besides, radiologists usually evaluate the risk of pulmonary nodules by interpreting the morphological characteristics on chest CT. But there is an overlap of morphological findings between malignant and benign nodules (11), as non-calcified granulomas also tend to present with malignant signs of lobulation or speculation (12, 13). Therefore, it is still a challenge for radiologist to differentiate lung cancers form benign solid pulmonary nodules.

Dual-energy computed tomography (DECT) has advantages in chest imaging by providing multiple quantitative parameter such as iodine concentration (IC) and effective atomic number (Zeff). It also can reduce the use of required contrast agent and the radiation dose by omitting a true unenhanced CT (14). Previous studies demonstrated that IC or normalized iodine concentration (NIC) could differentiate lung cancers from inflammation (15, 16) and differentiate malignant from benign solitary pulmonary nodules (17–19). All these studies only investigated the quantitative parameters that were related to iodine and enhancement, however, neglected the role of nodule size. Besides, solid and subsolid nodules were not separately examined in most studies (15–17, 19). One of them reported that 18 of 33 solid nodules were malignant while 16 of 16 subsolid nodules were adenocarcinomas (19), which was similar to the results of large cohorts (20, 21). Hence the DECT studies in subsolid nodules focused on the differentiation of invasiveness of adenocarcinomas rather than that between lung cancers and benign lesions (22, 23).

Therefore, this study aimed to investigate the ability of quantitative parameters of DECT for differentiation between lung cancers and benign lesions in solid pulmonary nodules, and compare their diagnostic performance with nodule size and Lung-RADS.

## Materials and methods

### Patients

This study was approved by the Institutional Review Board of Sichuan Cancer Hospital, and the written informed consent was obtained from all participants. A total of 580 consecutive pulmonary nodules were preliminarily enrolled from the Sichuan Cancer Hospital from April 2020 to November 2021. The inclusion criteria were as following: patients with dual-phase contrast-enhanced chest DECT, patients with solid pulmonary nodules (diameter < 3 cm), and histopathologic diagnosis *via* surgical resection. The exclusion criteria were as following: subsolid nodules (n = 414, 378 lung cancers and 36 benign lesions), with cancer history in previous 5 years (n = 2), receiving anti-cancer treatment prior to DECT (n = 10), unsatisfactory image quality due to respiratory and movement artifacts (n = 3).

**Figure 1:**
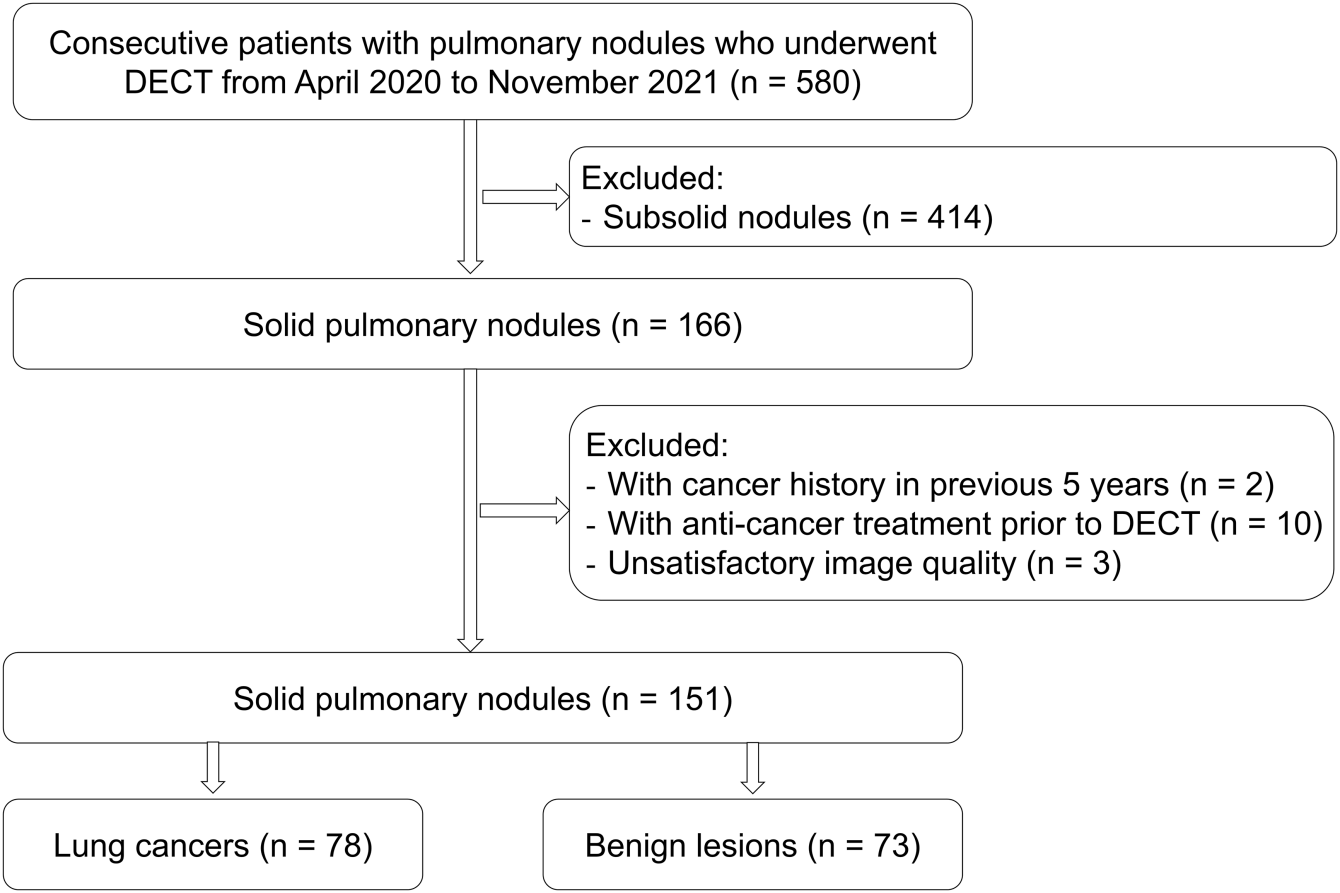
The flowchart for nodule recruitment. DECT, dual-energy computed tomography.

A total of 151 solid pulmonary nodules including 78 lung cancer (69 adenocarcinomas, 6 squamous cell carcinomas, and 3 small cell lung carcinoma) and 73 benign lesions (34 inflammations, 24 granulomas, 10 benign tumors, and 5 other benign entities) from 147 patients were finally enrolled in this study (**Table 1**) (**Figure 1**).

**Table 1:** The characteristics of solid pulmonary nodules.

Characteristics	Lung cancer (n=78)	Benign lesion (n=73)	*P*
Histologic subtype			
Adenocarcinomas	69		
Squamous cell carcinomas	6		
Small cell lung carcinomas	3		
Inflammations		34	
Granulomas		24	
Benign tumors		10	
Other benign entities		5	
Gender			0.194
Female	43	32	
Male	35	41	
Age (years)	57.9 ± 10.9	55.5 ± 11.3	0.189
Diameter (mm)	16.7 ± 6.1	14.2 ± 6.3	0.013
Lung-RADS (diameter)			0.008
2	2	4	
3	3	9	
4A	25	34	
4B or 4X	48	26	
Volume (cm^3^)	4.187 ± 4.160	2.869 ± 3.399	0.036
Lung-RADS (volume)			0.050
2	0	3	
3	5	6	
4A	22	30	
4B or 4X	51	34	
Zeff_A	8.07 ± 0.68	7.73 ± 0.49	0.001
IC_A (mg/mL)	1.43 ± 0.86	0.59 ± 1.43	< 0.001
NIC_A (%)	13.46 ± 8.25	5.54 ± 13.28	< 0.001
Zeff_V	8.42 ± 0.56	7.84 ± 1.01	< 0.001
IC_V (mg/mL)	2.03 ± 0.76	0.68 ± 0.97	< 0.001
NIC_V (%)	36.74 ± 13.49	11.92 ± 17.85	< 0.001

Granulomas are caused by mycobacterium tuberculosis, cryptococcus neoformans, and other unspecified conditions. Benign tumors include sclerosing pneumocytoma, hamartoma, and bronchial adenoma. Other benign entities include intrapulmonary lymph node and fibroplasia. Lung-RADS, Lung CT Screening Reporting and Data System; Zeff_A, effective atomic number in arterial phase; IC_A, iodine concentration in arterial phase; NIC_A, normalized iodine concentration in arterial phase; Zeff_V, effective atomic number in venous phase; IC_V, iodine concentration in venous phase; NIC_V, normalized iodine concentration in venous phase.

### Image acquisition

All DECT scans were performed on a second-generation dual-source CT (Somatom Definition Flash, Siemens Healthcare, Forchheim, Germany). A total of 90 ml contrast medium (370 mg iodine/mL, Iopromide, Bayer, Guangzhou, China) was injected *via* an antecubital vein at a flow rate of 3.0 mL/s, and followed by 30 ml of physiological saline at the same flow rate. The arterial phase was automatically triggered 5 s after the predetermined threshold (100 HU) was reached in a region of interest (ROI) that was placed at the ascending aorta at the layer of the pulmonary trunk. The venous phase was scanned 30 seconds after the arterial phase.

The same acquisition and reconstruction parameters in arterial and venous phases were used: tube voltage, 80/Sn140 kV; reference current, 205/87 mAs; pitch, 0.55; rotation time, 0.28 seconds; collimation, 64 × 0.6 mm; field of view, 350 × 350 mm; iterative reconstruction algorithm, SAFIRE (Strength level 4, Siemens Healthcare); reconstruction kernel, Q30f; matrix, 512 × 512; slice thickness, 0.5 mm; slice increment, 0.5 mm. Automated tube current modulation (CARE Dose 4D, Siemens Healthcare) was applied.

### Image analysis

The virtual non-enhanced image (VNI) was firstly obtained using arterial phase of DECT on a commercially available workstation (SyngoVia VB20, Siemens Healthcare). Second, all solid pulmonary nodules were automatically detected, segmented, and measured on the VNI using the uAI platform (United Imaging Healthcare, Shanghai, China), which is an artificial intelligence software based on deep learning method (24, 25). The segmentation results were assessed by two thoracic radiologists (JL and HQ, with 6 years and 11 years of experience) in the lung window (level - 500 HU, width 1500 HU). No manual adjustments of the segmentation results were conducted to avoid inter- and intra-observer variability, as all the segmentation results were satisfactory to both radiologists. Third, the diameter and volume were recorded. The diameter was the average of the maximal long-axis diameter and the perpendicular diameter on the maximum transverse plane of the nodule. The volume was calculated by multiplying the number of voxels by the unit volume of a voxel. Fourth, both radiologists (JL and HQ), who were blinded to histopathological results, were encouraged to categorize all the solid pulmonary nodules according to Lung-RADS (version 1.1) (26). As the category 4X required subjective assessment, the cases of disagreement between the two radiologists were resolved by consulting a third thoracic radiologist with 26 years of experience (PZ). All the solid pulmonary nodules were finally categorized into 2, 3, 4A, 4B, and 4X according to the Lung-RADS basing on diameter and volume respectively.

**Figure 2:**
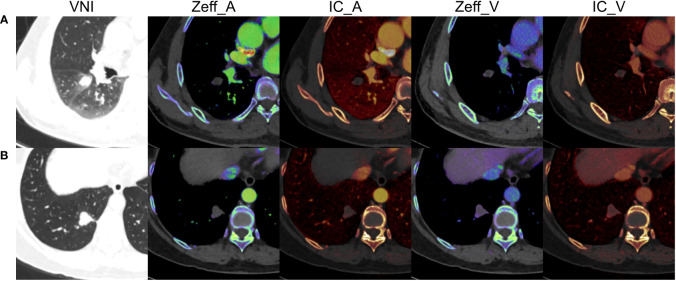
The representative dual-energy computed tomography images of solid pulmonary nodules. **(A)** A 49-year-old male with inflammation, diameter = 14.9 mm, volume = 1.949 cm^3^, Zeff_A = 7.57, IC_A = 0.10 mg/mL, NIC_A = 1.15%, Zeff_V = 7.80, IC_V = 0.50 mg/mL, NIC_V = 10.00%. **(B)** A 66-year-old female with adenocarcinomas, diameter = 18.8 mm, volume = 3.832 cm^3^, Zeff_A = 8.19, IC_A = 1.30 mg/mL, NIC_A = 10.92%, Zeff_V = 8.33, IC_V = 1.70 mg/mL, NIC_V = 27.42%. VNI, virtual non-enhanced images; Zeff_A, effective atomic number in arterial phase; IC_A, iodine concentration in arterial phase; NIC_A, normalized iodine concentration in arterial phase; Zeff_V, effective atomic number in venous phase; IC_V, iodine concentration in venous phase; NIC_V, normalized iodine concentration in venous phase.

The dual-phase DECT quantitative parameters were acquired on the same workstation. To minimize the variations caused by the patient’s circulation status, the circular ROIs were placed in the nodules and the aorta at the same layer on axial slice by a radiologist (PZ). The ROIs were drawn at the site that best characterized the nodules as large as possible on the axial slice showing the maximum diameter, avoiding necrosis and adjacent pulmonary vessels and bronchi. The Zeff, IC of nodule, and IC of aorta were measured. The NIC was calculated with the following formula: NIC = IC of nodule/IC of aorta × 100% (27). A total of 6 quantitative parameters were finally recorded, including Zeff in arterial phase (Zeff_A), IC in arterial phase (IC_A), NIC in arterial phase (NIC_A), Zeff in venous phase (Zeff_V), IC in venous phase (IC_V), and NIC in venous phase (NIC_V). The representative DECT images of solid pulmonary nodules are shown in **Figure 2**.

### Statistical analysis

Statistical analysis was performed using SPSS (version 25.0; IBM Corp., Armonk, N.Y., USA), Medcalc (version 18.2.1; MedCalc, Ostend, Belgium), and R (version 4.0.3; The R Foundation for Statistical Computing, Vienna, Austria). The categorical variables were analyzed using Fisher’s exact test, and the continuous variables were analyzed using independent sample *t*-test. Independent factors for differentiating lung cancers from benign solid pulmonary nodules were identified by inputting the significant variables using univariate logistic regression analysis. Then, multivariable logistic regression with backward stepwise selection and Akaike’s information criterion was applied to construct the combined model basing on these significant independent factors (28). The area under curve (AUC) of the receiver operating characteristic (ROC) curve was used to evaluate the diagnostic performance. The binomial exact method was used to determine the confidence interval (CI) of AUC. The optimal cutoff threshold was delimited according to Youden’s index of ROC analysis, and the corresponding sensitivity and specificity were also calculated. The DeLong test was used to compare the AUCs among the significant independent factors and the combined model (29). Further comparisons of sensitivity and specificity were performed using the McNemar test (30). A two-tailed *P*-value *<* 0.05 was considered statistically significant.

## Results

### Clinical characteristics and nodule size

No significant differences of gender (*P* = 0.194) and age (*P* = 0.189) were found between lung cancers and benign solid pulmonary nodules. The diameter and volume of lung cancers were higher than that of benign solid pulmonary nodules (*P =* 0.013 and 0.036). The Lung-RADS categorization of diameter was different between groups (*P* = 0.008) while that of volume was not (*P* = 0.050) (**Table 1**).

### Quantitative parameters of DECT

Lung cancers showed higher Zeff_A (8.07 ± 0.68 vs. 7.73 ± 0.49), IC_A (1.43 ± 0.86 vs. 0.59 ± 1.43), and NIC_A (13.46 ± 8.25 vs. 5.54 ± 13.28) than benign solid pulmonary nodules in arterial phase. (*P* = 0.001 or *P <* 0.001). In venous phase, lung cancers also showed higher Zeff_V (8.42 ± 0.56 vs. 7.84 ± 1.01), IC_V (2.03 ± 0.76 vs. 0.68 ± 0.97), and NIC_V (36.74 ± 13.49 vs. 11.92 ± 17.85) than benign solid pulmonary nodules (all *P <* 0.001) (**Table 1**).

### Univariate and multivariable analyses

Univariate logistic regression analysis showed that diameter, Lung-RADS categorization of diameter, volume, Zeff_A, IC_A, NIC_A, Zeff_V, IC_V, and NIC_V were independent factors for differentiating lung cancers from benign solid pulmonary nodules (all *P <* 0.05) (**Table 2**).

**Table 2:** Univariate and multivariable logistic regression analysis for predictive factors.

	OR (95% CI)	*P*	AUC (95% CI)	Sensitivity	Specificity	Cutoff
Univariate						
Diameter (mm)	1.069 (1.013 - 1.127)	0.014	0.626 (0.544 - 0.703)	0.603	0.658	> 15.3
Lung-RADS (diameter)	2.023 (1.279 - 3.200)	0.003	0.643 (0.561 - 0.719)	0.615	0.644	> 4A
Volume (cm^3^)	1.099 (1.004 - 1.202)	0.040	0.621 (0.539 - 0.699)	0.615	0.644	> 2.173
Zeff_A	3.542 (1.658 - 7.570)	0.001	0.721 (0.642 - 0.791)	0.641	0.753	> 7.98
IC_A (mg/mL)	2.684 (1.708 - 4.218)	< 0.001	0.821 (0.750 - 0.878)	0.705	0.822	> 0.95
NIC_A (%)	1.108 (1.056 - 1.162)	< 0.001	0.829 (0.760 - 0.886)	0.692	0.849	> 9.81
Zeff_V	4.929 (2.406 - 10.097)	< 0.001	0.764 (0.689 - 0.830)	0.897	0.630	> 8.03
IC_V (mg/mL)	6.860 (3.716 - 12.662)	< 0.001	0.891 (0.830 - 0.936)	0.987	0.753	> 0.95
NIC_V (%)	1.113 (1.076 - 1.151)	< 0.001	0.888 (0.826 - 0.933)	0.974	0.781	> 17.98
Multivariable						
Diameter (mm)	1.124 (1.040 - 1.214)	0.003	0.893 (0.832 - 0.937)	0.974	0.781	0.342
NIC_V (%)	1.117 (1.080 - 1.156)	< 0.001				

OR, odds ratio; AUC, area under curve; CI, confidence intervals; Lung-RADS, Lung CT Screening Reporting and Data System; Zeff_A, effective atomic number in arterial phase; IC_A, iodine concentration in arterial phase; NIC_A, normalized iodine concentration in arterial phase; Zeff_V, effective atomic number in venous phase; IC_V, iodine concentration in venous phase; NIC_V, normalized iodine concentration in venous phase.

Multivariable logistic regression showed the diameter and NIC_V were significant predicting factors (**Table 2**). The calculation formula for the combined model was as follows: ln (P/1−P) = - 4.473 + 0.117 × diameter + 0.111 × NIC_V, where P is the probability of lung cancer (cutoff > 0.342).

### Diagnostic performance comparison

**Figure 3:**
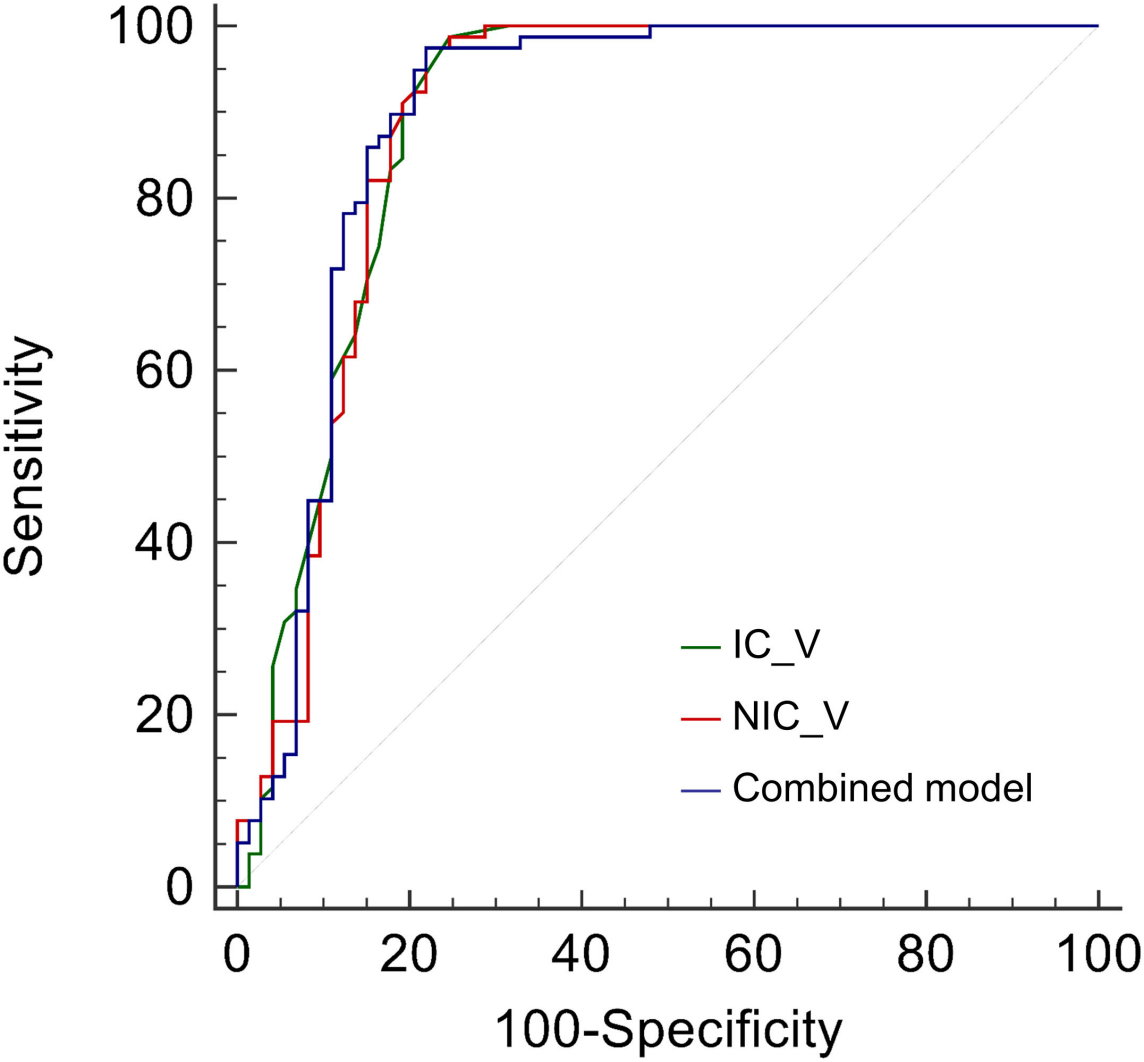
Receiver operating characteristic curves of iodine concentration in venous phase (IC_V), normalized iodine concentration in venous phase (NIC_V), and combined model for differentiating lung cancers from benign solid pulmonary nodules.

The IC_V, NIC_V, and combined model showed good diagnostic performance with AUCs of 0.891 (95% CI, 0.830 - 0.936), 0.888 (95% CI, 0.826 - 0.933), and 0.893 (95% CI, 0.832 - 0.937), and no significant differences of AUCs were found among them (**Table 3** and **Figure 3**). Using the cutoff values of 0.95 mg/mL, 17.98%, and 0.342, the IC_V, NIC_V, and combined model yielded excellent sensitivity (0.987, 0.974, and 0.974) and good specificity (0.753, 0.781, and 0.781) (**Table 2**).

**Table 3:** Comparisons of area under curves among predictive factors.

Predictive factors	*P*1	*P*2	*P*3
Diameter	< 0.001	< 0.001	< 0.001
Lung-RADS (diameter)	< 0.001	< 0.001	< 0.001
Volume	< 0.001	< 0.001	< 0.001
Zeff_A	< 0.001	< 0.001	< 0.001
IC_A	0.038	0.053	0.062
NIC_A	0.051	0.066	0.079
Zeff_V	< 0.001	< 0.001	< 0.001
IC_V	–	0.696	0.869
NIC_V	0.696	–	0.633
Combined model	0.869	0.633	–

P1 = P values between IC_V and the others; P2 = P values between NIC_V and the others; P3 = P values between combined model and the others. Lung-RADS, Lung CT Screening Reporting and Data System; Zeff_A, effective atomic number in arterial phase; IC_A, iodine concentration in arterial phase; NIC_A, normalized iodine concentration in arterial phase; Zeff_V, effective atomic number in venous phase; IC_V, iodine concentration in venous phase; NIC_V, normalized iodine concentration in venous phase.

The AUCs of IC_V, NIC_V, and combined model were higher than those of diameter, Lung-RADS categorization of diameter, volume, Zeff_A, and Zeff_V (all *P* < 0.001). The AUC of IC_V was also higher than that of IC_A (*P* = 0.038). There were no significant differences of AUCs between IC_V and NIC_A (*P* = 0.051), between NIC_V and IC_A (*P* = 0.053), between NIC_V and NIC_A (*P* = 0.066), between combined model and IC_A (*P* = 0.062), and between combined model and NIC_A (*P* = 0.079) (**Table 3**).

Further comparisons of sensitivity and specificity were performed between IC_V, NIC_V, combined model and IC_A, NIC_A. The results of McNemar test showed that the sensitivities of IC_V, NIC_V, and combined model were higher than those of IC_A and NIC_A (all *P* < 0.001), while there were no significant differences of specificities (all *P* > 0.05) (**Table 4**).

**Table 4:** Comparisons of sensitivity and specificity.

Comparisons	Sensitivity		Specificity	
	χ^2^	*P*	χ^2^	*P*
IC_V vs. IC_A	20.045	< 0.001	1.231	0.267
NIC_V vs. IC_A	17.391	< 0.001	0.364	0.549
Combined model vs. IC_A	16.000	< 0.001	0.308	0.581
IC_V vs. NIC_A	21.043	< 0.001	2.400	0.118
NIC_V vs. NIC_A	18.375	< 0.001	1.231	0.267
Combined model vs. NIC_A	16.962	< 0.001	1.231	0.267

IC_A, iodine concentration in arterial phase; NIC_A, normalized iodine concentration in arterial phase; IC_V, iodine concentration in venous phase; NIC_V, normalized iodine concentration in venous phase.

## Discussion

Our study explored the diagnosis performance of quantitative parameters of DECT and nodule size in distinguishing lung cancers from benign lesions in solid pulmonary nodules. The IC_V, NIC_V, and combined model consisting of diameter and NIC_V showed good diagnostic performance and outperformed the nodule size, Lung-RADS, Zeff_A, Zeff_V, IC_A, and NIC_A. The combined model did not increase the diagnostic performance compared with IC_V or NIC_V. These results indicated that the differentiation of lung cancers from benign lesions in solid pulmonary nodules was feasible using DECT-derived IC_V or NIC_V alone.

In recent years, DECT is an emerging diagnostic technology with various clinical applications, especially in thoracic imaging (31–33). IC, the most commonly used quantitative parameter of DECT, is considered to be equivalent to the actual value of enhancement. The enhancement of malignant nodules is associated perfusion and permeability of the capillaries, reflecting the underlying microvessel density and tumor angiogenesis (34, 35). The iodine parameters from DECT were significantly correlated with perfusion CT parameters with lower radiation exposure and contrast agent usage, which were considered surrogate measures for vascularity and perfusion (14, 36, 37). We found the lung cancers accumulated more iodine than benign solid nodules, which was consistent with previous reports (15, 17–19). Zeff quantitatively represents the composite atom for a compound or mixture of various materials (32). González-Pérez et al. found lower Zeff correlated with malignant pulmonary lesions, which was contrary to our result (38). The potential reason might be the different distribution of histologic subtypes in the included pulmonary lesions (39). Further study with large sample is needed to address this issue in the future.

This study also compared the diagnostic performance of iodine parameters and Zeff in differentiating lung cancers from benign lesions in solid pulmonary nodules. Our results showed that the IC_V and NIC_V had higher AUCs than Zeff_A and Zeff_V, and had superior sensitivities than IC_A and NIC_A, which was similar to previous studies (16, 17). Generally, the iodine contrast agent can easily leak into the intercellular space in lung cancer, due to angiogenesis, loose capillary endothelial cells, and incomplete basement membranes. Besides, the microvessels are tortuous in lung cancer, and the contrast agent flows slowly. In arterial phase, the microvessels cannot be full of the contrast agent, but the contrast agent can fill the microvessels and penetrate into the intercellular space in venous phase (40). Therefore, the IC_V was higher than IC_A in lung cancers (*t* = 7.919, *P* < 0.001) but not in benign lesions (*t* = 0.529, *P* = 0.598) in our study. Recent study of DECT also used radiomic features from virtual monoenergetic image to differentiate benign from malignant pulmonary nodules (41). However, the complexity of this approach limited its integration into the clinical workflow as it required additional software (37).

The Lung-RADS (version 1.1) introduced volume to stratify the malignant risk of pulmonary nodule (42–44). Thus both the Lung-RADS of diameter and volume were used to categorize the solid pulmonary nodules in this study. The AUCs of nodule size and Lung-RADS categorization ranged from 0.621 to 0.643, and were lower than that of IC_V and NIC_V. These results indicated that the nodule size and Lung-RADS categorization had inadequate diagnostic efficiency. Besides, the combined model integrating nodule diameter and NIC_V did not significantly improve the diagnostic efficiency compared with IC_V or NIC_V. Therefore, using the IC_V or NIC_V alone enabled diagnostic utility in the differentiation between lung cancers and benign lesions in solid pulmonary nodules.

The current management guidelines of pulmonary nodules in lung cancer screening recommend follow-up CT at 3 months, positron emission tomography/computed tomography (PET/CT), or tissue sampling for solid nodules over 8 mm (7, 26, 45). Although PET/CT provides more metabolic information than CT alone, this modality is associated with excessive radiation dose and high cost. The transthoracic needle biopsy and bronchoscopy, as invasive tissue sampling approaches, are often selected based on location of the nodule, clinical expertise, comorbidities, and physical condition of patients. Previous studies showed transthoracic needle biopsy had a higher pooled diagnostic than bronchoscopy, but was associated with an increased risk for pneumothorax and hemorrhage (46, 47). Therefore, the DECT may be alternative in the follow-up CT for further assessment of solid pulmonary nodules as the IC_V and NIC_V have diagnostic utility in distinguishing lung cancers from benign lesions.

There are several limitations in this study. First, this was a single center study with a relatively small sample size, further external validation datasets are needed to test the replicability of our results. Second, the nodule size was assessed using VNI other than true non-enhanced image. A phantom study of lung tumor model found that VNI could be alternative to true non-enhanced image in volumetry (48). Third, comprehensive morphological characteristics were not included in this study. The combination of morphological and quantitative features may improve the diagnostic performance, and further study is needed. Fourth, we did not study the intermediate nodules separately and the subsolid nodules were also excluded. The adding value of the IC_V and NIC_V to those nodules and Lung-RADS needs more researches.

In conclusion, the DECT-derived IC_V and NIC_V had good diagnostic performance in differentiation of lung cancers from benign lesions, and could be a non-invasive biomarker to predict malignant risk of solid pulmonary nodules in clinical practice.

## Data availability statement

The original contributions presented in the study are included in the article/supplementary material. Further inquiries can be directed to the corresponding author.

## Ethics statement

The studies involving human participants were reviewed and approved by the Institutional Review Board of Sichuan Cancer Hospital. The patients/participants provided their written informed consent to participate in this study.

## Author contributions

CH, JL, and PZ conceived and designed the study. YL, LL, HQ, LG, and SH collected the data. CH and JL analyzed the data and drafted the manuscript. All authors reviewed the manuscript and PZ revised the final manuscript. JL, LG, and PZ provided funding for the study. All authors contributed to the article and approved the submitted version.

## Funding

This study was supported by the National Natural Science Foundation of China (grant number 82202141), the Sichuan Science and Technology Program (grant numbers 2021YFS0075, 2021YFS0225), the Chengdu Science and Technology Program (grant number 2021-YF05-01507-SN), and the Beijing Medical Award Funding (grant number YXJL-2022-0105-0143).

## Conflict of interest

The authors declare that the research was conducted in the absence of any commercial or financial relationships that could be construed as a potential conflict of interest.

## Publisher’s note

All claims expressed in this article are solely those of the authors and do not necessarily represent those of their affiliated organizations, or those of the publisher, the editors and the reviewers. Any product that may be evaluated in this article, or claim that may be made by its manufacturer, is not guaranteed or endorsed by the publisher.
